# Interactive Crossword Puzzles as an Adjunct Tool in Teaching Undergraduate Dental Students

**DOI:** 10.1155/2022/8385608

**Published:** 2022-05-23

**Authors:** Abubaker Qutieshat, Nutayla Al-Harthy, Gurdeep Singh, Viresh Chopra, Rayhana Aouididi, Rayan Arfaoui, Sapna Dileesh, Ahmed Alsadoon, Omaima Al-Nadhiri, Shima Al-Busaidi, Sumaiya Al-Rashdi, Mohammad S Alrashdan

**Affiliations:** ^1^Adult Restorative Dentistry, Oman Dental College, Muscat, Oman; ^2^Member of Staff & Honorary Researcher, Dundee Dental Hospital & School, Dundee, UK; ^3^Oral Medicine & Oral Surgery, Jordan University of Science & Technology, Irbid, Jordan; ^4^Jordan University of Science & Technology, Irbid, Jordan; ^5^English Department, Oman Dental College, Muscat, Oman; ^6^Oman Dental College, Muscat, Oman; ^7^Oral & Craniofacial Health Sciences, College of Dental Medicine, University of Sharjah, Sharjah, UAE

## Abstract

**Background:**

In the restorative dentistry module of the undergraduate dental program, dental students encounter many new terms and concepts in a short period of time. The provision of adequate learning support to reinforce key concepts can be challenging.

**Aim:**

The purpose of this study is to determine student perceptions of how effective interactive crossword puzzles are as adjunctive tools to enhance the learning of restorative dentistry.

**Methods:**

Students completed interactive crossword puzzles created by the authors, with a reward awarded to the first group of students who completed the puzzles successfully. The interactive crossword platform was programmed using the ReactJS framework and designed using Tailwind CSS. An evaluation of the student's perception of this educational method was conducted using textual feedback and Likert-scale questionnaires.

**Results:**

Students found the crossword puzzles engaging, meaningful, and successful as indicated by their favorable Likert scores and feedback. Written comments revealed student enthusiasm for and a desire to be exposed to more of these exercises.

**Conclusions:**

This work sheds light on the potential advantages of incorporating interactive crossword puzzles into the restorative dentistry course from a student's perspective. The crossword puzzles improved students' ability to review and reinforce concepts and terminology and proved to be meaningful and enjoyable. The web-based nature of the tool ensured good student responsiveness and engaged the entire class in an interactive, competitive setting. Application of the hint option, which offered a text of relevant reading material, helped students understand, retain more knowledge, and engage with course material more than they might have otherwise.

## 1. Introduction

The use of active learning strategies is recognized as good practice in undergraduate education and is now a widely accepted tool for information delivery and retention [1]. Over the past two decades, various forms of active learning methods have been proposed with the goal of enhancing active engagement with the material and help students retain new knowledge.

The application of active learning tools is commonly done with several aims in mind: provide an enjoyable learning experience; increase student motivation to change studying habits; develop new relationships among concepts; build on students' prior knowledge; and increase student interest in course material [1]. As an active learning tool, crossword puzzles are especially helpful in teaching details that need to be memorized [2].

Certain cognitive processes, such as memorizing and recalling, in the learning of restorative materials are often perceived as time consuming and frustrating, particularly when dealing with new terminology and phrases. Using memorization and recall strategies, often referred to as passive learning, may lead to greater difficulty in integrating new knowledge with prior learning and in applying new theoretical concepts in relevant contexts [3].

During freshman and sophomore years, it is inevitably difficult for dental students to learn and recall the jargon that is mostly unknown to them especially when they start to learn about modern dental concepts of applied materials science. Many new terms and concepts have been introduced to the dental curriculum in recent years, which could be associated with either alternative definitions or competing theories between conventional dentistry and the more “modern” minimally invasive dentistry. This has posed a challenge in maintaining adequate conductive environment and learning activities for students to reinforce key concepts.

New educational technologies and teaching methods are increasingly being used in dental education to supplement the delivery of learning resources. In our department, the Department of Adult Restorative Dentistry, there has been increasing interest in dental educational strategies in the past five years, with the development of new concepts and adjunctive tools such as problem-based learning [4], student-centered learning [5], and self-directed clinical skill acquisition [6]. Given the success of those approaches in our educational setting, the implementation of a game, as an adjunctive tool in dental education, could provide an extra dimension of information that could certainly fine-tune our educational strategies and methodologies.

The use of games in education can be helpful in guiding the process of teaching to good effect especially among growing tech-savvy learners. By utilizing games carefully, educators can direct their teaching environment toward success in raising engagement, motivation, and performance [7]. Although advocated by many, the integration of games in dental education has only started to gain traction in the last few years [8–10]. It is in this context that the idea of implementing an interactive crossword puzzle game arises in this article.

The potential for crossword puzzles to help students overcome their learning difficulties has been scarcely documented within dental education literature [11]. However, the use of crossword puzzles as an active learning tool in medical education has been reported abundantly during the past two decades [12–17]. As part of a curricular development effort for undergraduate dental students, this work describes an adjunctive teaching method whose main focus is to develop exploratory thinking, in order to enhance motivation toward the learning of restorative dentistry. The findings we present here address the research question: whether the use of interactive crossword puzzles can be successfully introduced in the course and—if suitable—to enhance the learning of restorative dentistry.

### 1.1. Theoretical Framework

The information processing theory may be used to rationalize issues concerning the acquisition of scientific terminology and its appropriate use in problem-solving [18]. Efficient cognitive processing is associated with the focused nature of information processing as well as the ability to complete tasks quickly and efficiently. This can be accomplished through repetition and the deliberate allocation of limited cognitive resources to the most relevant information [19].

Game-based learning has been proven to enhance learning motivation, improve educational outcomes, and even reduce anxiety [20–22]. These factors, taken together, stimulate learners' sense of competition and motivation for cooperative behavior in order to get rewards. Simultaneously, Csikszentmihalyi's flow theory demonstrates that when students are actively involved in an experience, they will often be more immersed and more attentive to the task [23]. Given that the constructivism theory emphasizes knowledge building through problem-solving and social interaction [24], the practical application of constructivist game-based strategies is dependent on the quality of the evidence demonstrating their efficacy and the recognition of said pedagogical strategy. It is therefore evident that more constructivist game-based learning environments are required [25]. In order to create interactive learning platforms that engage students in meaningful learning and help them develop satisfactory problem-solving skills, educators must be involved in the design process [26].

It is critical to determine how students can achieve a balance between the difficulties they face and their skill level when teaching clinical and procedural dental skills. According to current research, achieving this balance is dependent on the student's access to various auxiliary resources such as evidence-based and evidence-informed practice especially when the use of scholarly search engines is not explicitly spelled out in the dental curriculum [27, 28]. Much of the literature contends that effective individualized learning employs educational resources in such a way that students get what they need, when they need it [29]. As a result, a personalized assistance strategy would provide the processes and mechanisms required to complete academic tasks in educational scenarios that are game-based. This would assist students in meeting their objectives, achieving proper balance, and improving their learning experiences and outcomes.

While information processing and the constructivism theories account for the cognitive processes involved in learning, Ausubel's assimilation learning theory provides an affective and behavioral perspective by taking into account students' attitudes toward and motivation for learning [30]. It specifically proposes important conditions required for the successful acquisition of deep learning: for new information to be learned meaningfully, it must be connected to the learner's prior knowledge, be relevant, and be actively integrated into the learner's cognitive structure. As a result, this theory emphasizes the learner's central role in knowledge construction and distinguishes between the mere act of memorization and meaningful learning, which is particularly relevant to the current study [31].

## 2. Methods

Ethical clearance to conduct this study was obtained from the Research and Ethics Committee (REC) of Oman Dental College (ODC).

### 2.1. Interactive Crossword Puzzle Design

The interactive crossword platform (CrossLearn) was programmed using the ReactJS framework and designed using Tailwind CSS. Performance data storage and transmission were achieved using firebase and cloud integrations. In recognition of the usefulness of CrossLearn, the project team has made the platform freely available at https://cephcad.com/crosslearn [32]. The platform is rendered entirely on the client side in order to reduce the volume of server-side traffic and associated costs. User experience, ease of use, and enjoyment were the main design considerations during the application's development. The puzzle timer, statistics, and the number of hints utilized are calculated and stored in the backend server memory and are later displayed to the user.

**Figure 1:**
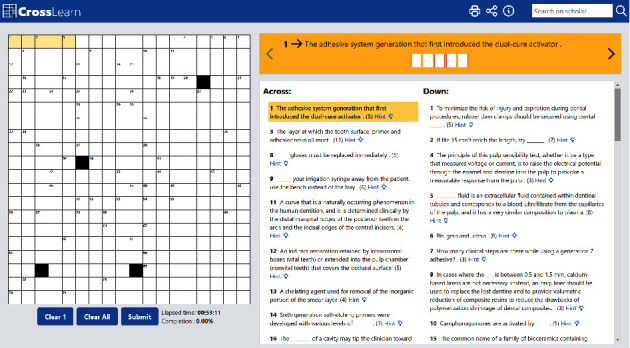
Interactive crossword puzzle design. The puzzles consist of horizontal and vertical columns, with the words running horizontally from left to right and vertically from top to bottom.

**Figure 2:**
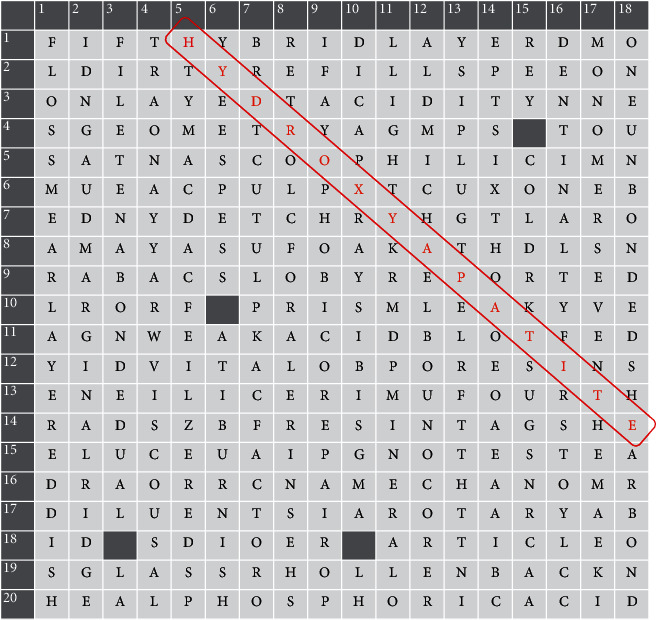
For additional competitiveness, each puzzle contained a hidden diagonal word that must be identified in order to complete the challenge. In this example, the hidden word is “hydroxyapatite.”

**Figure 3:**
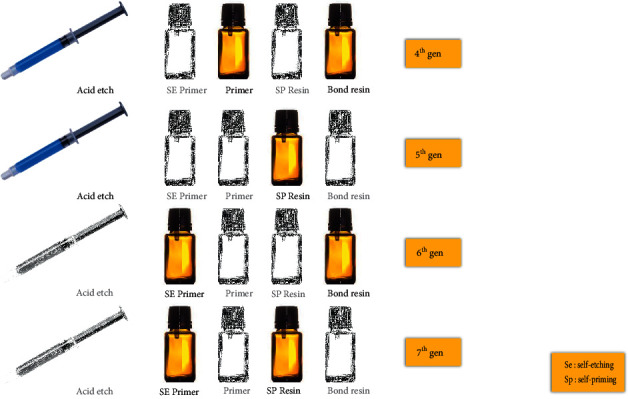
There was a hint for each question, which included snippets from lecture slides, scholarly articles, and textbooks. In the figure is a hint for Question 1 (across): “The adhesive system that first introduced the dual-cure activator.”

The puzzles contain both vertical and horizontal columns with the words running horizontally from left to right and vertically from top to bottom (Figure 1). In addition, there was one hidden diagonal word in each puzzle that must be identified to complete the challenge and to add some additional competitiveness (Figure 2). Students were asked to formulate a meaningful paragraph to relate the hidden word or phrase to the course material. A mixture of topic-specific and general dental terms and phrases was used to maintain a moderate difficulty level. The set of words and phrases of focus for each topic were discussed among departmental faculty members prior to their incorporation into the puzzle. Hints were provided for each question and included snippets from lecture slides, scholarly articles, and textbooks that relate to the correct answer (Figure 3). The Google Scholar search box was made available in each puzzle to encourage students to use scholarly research databases and search for papers with relevant keywords directly. Although a timer is set to start when the game begins, there was no time limit, and students could take as long as they needed to look into hints and answer the questions. This exercise was to be completed over a period of one week in a collaborative and cooperative manner in groups of no more than three students. An emphasis was placed on enhancing the competitive aspect of this exercise, and a reward was given to the first team to correctly complete the puzzle. When more than one group is able to solve the puzzle in a comparable manner (e.g., more time and less hints cf., less time and more hints), the group that finds the hidden word first is considered as the winning group.

Hints were designed carefully to include keywords that imply important concepts and links, phrases and terms that are important to memorize, and relevant clinical scenarios. The hints and answers were linked to specific learning objectives to ensure content validity. The hints were carefully constructed around keywords that indicated critical concepts that require a thorough understanding (e.g., resin-dental substrate interaction), as well as certain phrases and terms that must be retained (e.g., hybrid layer, resin tags).

An example is provided in Figure 1; this crossword was used to review and reinforce terminology and concepts on adhesive dentistry including (1) basic concepts in adhesion and the rationale for bonding mechanisms; (2) the chemical composition of adhesives and how various monomers and other components contribute to the process of adhesion; (3) techniques for successfully bonding to various substrates; and (4) clinical applications of adhesives.

With the goal of maintaining a moderate difficulty level as well as including some simple questions, each crossword puzzle had about one-fourth of its questions pertaining to a broader set of dental concepts and terms that were previously covered throughout the course (e.g., health and safety, diagnosis, vital pulp therapy, root canal treatment, direct and indirect restorations). Four departmental faculty members reviewed the content and format of the questions.

### 2.2. Evaluation of Intervention

A questionnaire was administered to a cohort of undergraduate 4th-year dental students (*n* = 67) which included specific questions regarding the use of interactive crossword puzzles within the classroom as a learning intervention. The questionnaire's purpose and delivery date were communicated to students in a preliminary e-mail. When the winning team was announced, the students were notified that the questionnaire had opened. One week after the questionnaire opened, reminders were sent to those who had not yet completed it. After being open for two weeks, the survey was closed.

Students were asked to rank question statements using a Likert scale (five responses ranging from “strongly agree” through “neutral” to “strongly disagree”). The questionnaire was developed to assess the effectiveness of this learning intervention based on its overall educational objective. A similar Likert questionnaire was previously tested and proven to be reliable [4, 5]. Students were asked to rate six broad areas of their perceptions of the interactive crosswords for enjoyability and satisfaction, knowledge reinforcement, learning key concepts and terminology, efficacy as a novel learning tool, the competition factor, the collaboration factor, and the interest in incorporating this tool into other courses. This was piloted upon a convenience sample of postgraduate students who had completed the crossword puzzles. The six Likert-scale questions are listed in Table 1. Feedback in writing was also collected at the end of the module. The questionnaire and feedback were collected anonymously, and data were analyzed by Microsoft Excel version 21 using descriptive statistics.

## 3. Results

For the example presented in this paper, ten groups (of a total of 24 groups, each with 2–3 students) completed the interactive crossword puzzle among which only two groups managed to identify the hidden word (i.e., hydroxyapatite). On average, it took the students about 9 hours and 60 hints to solve the puzzle. Based on the performance of all the groups (number of hints used and time taken to complete the task), one group was selected as the winner. The winning group received a certificate of achievement from the department. The progress toward completion was 74.9% (SD 23.1%) for those who failed to complete the puzzle.

### 3.1. Questionnaire Data

Of the 67 students in this course, 94% (63) were local students and 6% (4) international students. Students were all females with ages ranging from 23 to 26 years. The mean age was 23.5 years, and the median age was 23 years. The course of study for these students already incorporates a number of learning technologies, which are well supported by an institution-based e-learning support department.

**Figure 4:**
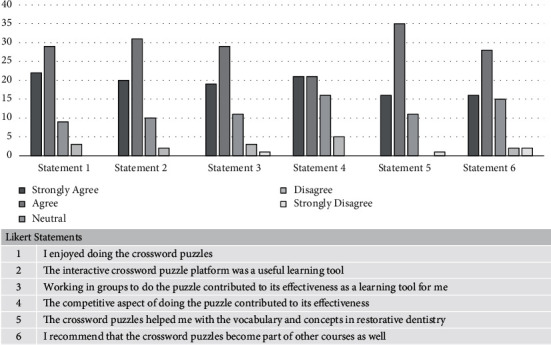
Students' responses to the questionnaire on their perceptions about the interactive crossword puzzle.

A total of 63 students responded to the questionnaire (94% response rate). The internal consistency of Likert responses was good (Cronbach's alpha 0.84). Analysis of the data from the questionnaire revealed a high level of student satisfaction. A summary of the results is presented in Figure 4.

### 3.2. Student Feedback

Forty-seven students made comments. As the majority of these comments were brief, a detailed thematic analysis was not performed; however, all participants' comments were positive and reflective of notions of satisfaction, enjoyment, motivation, collaborative learning, and knowledge retention.

Some students have hinted that they initially were not interested in games within learning contexts, but that after they experienced what it entailed, they gradually became attracted to it and eventually were able to thoroughly enjoy it.

*“I've always enjoyed a good chess puzzle but I never thought that partaking in a crossword puzzle as part of a university course would interest me, and yet I recall the time I spent trying to solve the puzzle as the most exciting and academically rewarding part of that semester.”—*Student #37.

One of the things that has stood out most to some students about the activity is that it is able to reduce the stress that comes with studying a difficult subject.

“*This activity alone played a crucial role in taking my understanding of the restorative dentistry course to the next level in no time. What made the crossword so appealing was that it did not look or feel like an act of strenuous studying, it did not reflect the burden of an assignment, nor did it resemble the fear and anxiety of an exam. It was simply a fun challenge and a hack to quickly be informed of the entire material without being too aware of the fact that one is actually ‘studying'*.”—Student #11.

There have been quite a few comments that emerged around the notion of motivation. Students were keen on acquiring additional knowledge in a nonformal learning environment which was perceived by the authors of this work as particularly interesting and an indicator of success.

“*Like many others, the crossword did the trick effortlessly for me. I was studying ahead to later spot answers for hints, and sometimes I did the reverse by taking a hint and setting out to decipher it by quickly skim-reading any relevant article that I could lay my hands on, only because I did not want to miss anything that would leave a set of squares blank in the puzzle. And by doing so, I eventually ended up with deep and detailed comprehension of numerous topics with ease*.”—Student #25.

*“Getting stuck a few times only fueled an ongoing motivation to see the puzzle complete, I admit to neglecting a couple of other courses for a few days at the expense of unlocking one difficult riddle at some point only because it was that entertaining and satisfying to figure it out, I was hooked.”*—Student #4.

In terms of retaining and reinforcing key concepts and terminology, students also recognized that improving grades, without additional written or verbal feedback, over a period of time allowed them to see positive progress and boosted their confidence.

“*What I noticed was that any definition, explanation, process, material or procedure I actively searched for in the aims of decoding another riddle, would permanently be embedded in my long-term memory, meaning that I never need to go back to that piece of information and read it again.”*—Student #49.

*“In terms of adjunct learning tools, over the years I've tried group work projects, PowerPoint presentations, team debates and essay assignments and nothing comes close to the benefits I gained from crossword puzzles, I'd choose it over anything else every time.”*—Student #29.

Within the context of collaborative, small group learning, the students talked extensively about the value of adding a competitive element into the activity. In some instances, this went as far as suggesting that games should become part of the assessment process.

“*Having a deadline of “whoever solves it first” was an excellent touch. I found colleagues pairing up in the hopes of completing the puzzle faster. In my opinion, it built comradery and a much healthier competitive atmosphere in comparison to that of competing over an exam grade, which made the puzzle much more encouraging to attempt. I wish our exams had that “fun” element!”*—Student #5.

Within the context of game design, several students stated that the design of the game was valued for its built quality and all the features it offered. This included carefully tailored hints, easy access to Goggle Scholar, and a dashboard that shows progress and provided an additional competitive dimension to the game.

*“The puzzle itself and how it's generated plays a huge role in the experience. The first puzzle for instance was easy and fun, but this was an absolute delight. The harder the puzzle the better it was and the more creatively written the riddles were the more enjoyable*—*the “hydro-xyapatite” “hydro-phobic” “hydro-philic” part was no short of genius, and it was probably my favorite riddle out of them all.”*—Student #5.

The transferability of the game to other courses was perceived as an essential attribute; this was of particular relevance to the more difficult topics. Some students recognized that, whenever new challenging concepts and terminology arise, incorporating games into the curriculum could be valuable.

*“I, without doubt, believe I could've boosted my progress in other courses had they implemented this activity or something similar in their curriculum as well. As a student who often struggles with motivation, to rekindle a desire to learn amidst traditional mundane teaching methods and who excels when given a creative challenge, I cannot possibly praise or recommend crossword puzzles enough for any educational purpose at any level.”*—Student #62.

## 4. Discussion

There has been an increase in research in the field of game-based learning in recent years with its benefits becoming more established and widely recognized in higher education settings. According to a systematic review [33], game-based learning is rapidly gaining acceptance and traction in higher education. A variety of benefits were overwhelmingly endorsed by students and educators in higher education, including student engagement, motivation, and enjoyment [34, 35]. In the present study, the incorporation of interactive crossword puzzles into the undergraduate dental curriculum was reported to be a valuable adjunctive learning tool; students found the activities helpful in reinforcing their knowledge, contributing to their learning, and engaging them in a collaborative and competitive manner.

Providing small groups of two or three with this method would fit within the broad domain of small group learning, requiring students' participation and interaction and reflecting more of a student-oriented rather than a teacher-focused approach [36].

Learning in small groups leads to a greater level of academic achievement and a more favorable attitude toward learning, and promotes deeper understanding, better participation, better problem-solving skills, and improved interpersonal relationships [37]. The effects of implementing interactive crossword puzzles are likely to be similar, and our findings indicate that students expressed more positive views regarding the course and its topics.

The answers to the questions were specifically chosen to relate to the topic and serve as anchors for key concepts and terminology covered in the course. In a quick and informal manner, the game provides students and instructors immediate feedback about any misconceptions or misunderstandings. As any other game, the end point will be achieved only as a result of a continuous play, exploring the available hints and experimenting with new variations. The already completed words will therefore provide additional context for the remaining incomplete entries, enhancing the game's immersion and pedagogical value. This is in line with the findings of other researchers on game-based learning, which have found that such game designs can boost student engagement and ensure that the anticipated learning outcomes are met [38, 39].

The game design also offered students the option to integrate information from scholarly articles by using the embedded Google Scholar search box and also helped establish their understanding of the evidence-based scientific literature. There have been multiple calls for integrating research-based material into the undergraduate curriculum [40–42]. It is therefore important for educational institutions to devise strategies that prepare students to be able to read research papers and appraise evidence from various research sources [43]. Incorporating a Google Scholar box into the game offered students an opportunity to obtain a clearer understanding of whether the information provided to answer a question is correct when cross-referenced to scholarly sources, a practice that is strongly advocated in clinical decision-making [44]. Advocates of crosswords assert that these puzzles promote healthy skepticism toward unverified information and unquestioned assumptions serving as a platform for the development of problem-solving skills, critical thinking, decision-making, and deductive reasoning [45].

According to several studies, medical and dental students' communication skills do not appear on par with what is expected of them and may even degrade as they progress through their training [46–48]. It was intended that the activity described herein would also be a venue for improving students' interpersonal skills, which in turn would motivate and stimulate their learning. It has been shown that a classroom environment that fosters cooperation and teamwork enhances learning in medical and dental education [36, 49].

This activity includes a competitive element, particularly the possibility of competing against peers who consider themselves academic equals. There were approximately 8% of students who did not agree with this element; this may be attributed to their learning styles and their vulnerability when being put under additional pressure. The interactive crossword puzzles provided students with the opportunity to feel challenged, excited, and encouraged to revise relevant material collaboratively with their peers. Essentially, this is in keeping with our philosophy that improving knowledge of cooperation and teamwork as well as acquiring the ability to successfully apply these skills in a demanding context is so fundamental in dental education.

Although the potential of interactive games in dental education is huge [9], it is surprising that only a few studies to date have been concerned with exploring its potential within the dental curriculum [50, 51]. The main reason for this is that tutors may not have the time to develop these games on their own without expert support, not to mention the amount of time required to construct topic-specific content. Therefore, it may be helpful to establish a multidisciplinary group composed of dental educators, programmers, and game developers if games of this nature are to be added to the dental curriculum.

The fact that there were no negative comments from students and that some students made very positive comments about the activity is also considered evidence that the use of interactive crossword puzzles as an adjunct tool in learning was acceptable and effective.

As most of our students live off-campus and there is limited transport available, students would stay around the college campus until late hours which makes it difficult for them to fully engage in conventional forms of learning activities. Interestingly, the interactive crossword puzzles achieved an unintended goal of lightening up the after-hours atmosphere by providing both collaborative and competitive pedagogical elements outside the classroom setup.

Students were offered puzzles with varying levels of difficulty according to the amount of information and complexity of the topic (e.g., easy with ten questions, medium with ten to fifty questions, and hard with more than fifty questions). This also helped avoid familiarity with the task, ensuring the cognitive load remained high.

Ideally, student satisfaction data cannot be used to assess students' recall of essential concepts. It is evident, however, that the overall completion of the puzzle presented herein averaged 74.9% by the time the competition ended which, in turn, demonstrates good performance in recalling the content, favorable cognitive load, and increased cognitive capacity [52]. Within the context of advanced knowledge acquisition and transfer, there are two types of transfer, near and far, which describe the extent to which learners can apply their new knowledge [53]. The use of a crossword puzzle may not be appropriate in evaluating far transfer of knowledge; however, it is an excellent tool for evaluating near transfer [54, 55]. Students' performance in the game, therefore, is the evidence that they were able to recall most concepts and terminology set for them to achieve the anticipated learning objectives as well as perform near-transfer memory tasks favorably.

This study used a self-reported questionnaire as a data collection tool that is subject to an inherent limitation of subjectivity. One more limitation of the study is that it did not use a control group which limits the interpretation that students' positive perceptions arose from the intervention alone. All students expressed an interest in participating in the study, and given the length and complexity of the taught module, it was determined through discussions with school administrators that denying a control group access to the adjunctive learning tool might result in feelings of disappointment and resentment among students. In addition, the study was set to probe into students' perceptions on the effect of interactive crossword puzzles on their learning experience relative to the *status quo ante* of no game-based learning in the dental curriculum. Further work would be required to confirm the generalizability and applicability of the findings of this study to other dental schools. Such educational gaming platforms must be carefully designed and thoroughly thought through to ensure that all learning objectives are met and to increase the chances for success both technically and educationally.

## 5. Conclusions

In this work, we created a series of interactive crossword puzzles as an adjunctive teaching tool to help dental students reinforce concepts and terminology related to restorative dentistry. As reflected by the textual feedback and the favorable Likert ratings, the interactive crossword puzzle format offered students a much-needed opportunity to joyfully engage in active learning and promote the comprehension and retention of difficult material. Students have formed teams, collaborated, competed, and also applied their critical thinking skills to discuss relevant core concepts from the instruction, and justify their answers by referring to scholarly sources. Furthermore, students became more fluent in topic-specific terminology and more proficient in organizing thought processes that are well aligned with the predetermined learning objectives.

This work provides insight into the potential advantages of utilizing interactive games in dental education to reinforce concepts and terminology in an interactive and technologically rich learning environment. The carefully designed platform format ensured good student responsiveness and engaged the entire class in active learning. We believe that games in dental education have a great deal of potential to contribute to science- and evidence-based dentistry teaching and will take dental graduates to a higher level of intellectual development. More broadly, this study is an open invitation to other dental educators interested in active learning to test, improve, and comment on the activity presented here and to share their experiences with the scientific community.

## Figures and Tables

**Table 1:** Likert-scale statements. Five responses were available for each statement: “strongly agree,” “agree,” “neutral,” “disagree,” and “strongly disagree.”

Likert-scale statements	
1	I enjoyed doing the crossword puzzles
2	The interactive crossword puzzle platform was a useful learning tool
3	Working in groups to do the puzzle contributed to its effectiveness as a learning tool
4	The competitive aspect of doing the puzzle contributed to its effectiveness
5	The crossword puzzles helped me with the vocabulary and concepts in restorative dentistry
6	I recommend that the crossword puzzles become part of other courses as well

## Data Availability

Data are available on request from the corresponding author.
